# Functional characterization of two clip domain serine proteases in innate immune responses of *Aedes aegypti*

**DOI:** 10.1186/s13071-021-05091-9

**Published:** 2021-11-24

**Authors:** Hao-Cheng Wang, Qiu-Hui Wang, Biswajit Bhowmick, Yi-Xun Li, Qian Han

**Affiliations:** 1 Laboratory of Tropical Veterinary Medicine and Vector Biology, School of Life Sciences, Hainan University, Haikou, Hainan 570228 People’s Republic of China; 2 One Health Institute, Hainan University, Haikou, Hainan 570228 People’s Republic of China

**Keywords:** Innate immunity, *Aedes aegypti*, Prophenoloxidase, RNA interference, Clip domain serine proteases, Microbial infection

## Abstract

**Background:**

Clip domain serine proteases (CLIPs), a very diverse group of proteolytic enzymes, play a crucial role in the innate immunity of insects. Innate immune responses are the first line of defense in mosquitoes against the invasion of pathogenic microorganisms. The Toll pathway, immunodeficiency (IMD) pathway and melanization are the main processes of innate immunity in *Aedes aegypti*. CLIPS are classified into five subfamilies—CLIPA, CLIPB, CLIPC, CLIPD, and CLIPE—based on their sequence specificity and phylogenetic relationships. We report the functional characterization of the genes that code for two CLIPs in *Ae. aegypti* (*Ae*):* Ae-CLIPB15* and* Ae-CLIPB22*.

**Methods:**

Clustal Omega was used for multiple amino acid sequence alignment of *Ae-CLIPB15* and *Ae-CLIPB22* with different* CLIP* genes from other insect species. The spatiotemporal expression profiles of *Ae-CLIPB15* and *Ae-CLIPB22* were examined. We determined whether *Ae-CLIPB15* and *Ae-CLIPB22* respond to microbial challenge and tissue injury. RNA interference (RNAi) was used to explore the function of *Ae-CLIPB15* and *Ae-CLIPB22* in the defense of *Ae. aegypti* against bacterial and fungal infections. The expression levels of nuclear factor kappa B (NF-κB) transcription factors *REL1* and *REL2* in the Toll pathway and IMD pathway after bacterial infection were investigated. Finally, the change in phenoloxidase (PO) activity in *Ae-CLIPB15* and *Ae-CLIPB22* knockdown adults was investigated.

**Results:**

We performed spatiotemporal gene expression profiling of *Ae-CLIPB15* and *Ae-CLIPB22* genes in *Ae*. *aegypti* using quantitative real-time polymerase chain reaction. These genes were expressed in different stages and tissues. The messenger RNA (mRNA) levels for both genes were also up-regulated by Gram-negative bacteria *Escherichia coli*, Gram-positive bacteria *Staphylococcus aureus* and fungal *Beauveria bassiana* infections, as well as in the tissue injury experiments. RNAi-mediated knockdown of *Ae-CLIPB15* led to a significant decrease of PO activity in the hemolymph of *Ae. aegypti*, while other RNAi experiments revealed that both *Ae-CLIPB15* and *Ae-CLIPB22* were involved in immune defense against bacterial and fungal infections. The mRNA expression of NF-κB transcription factors *REL1* and *REL2* in the Toll pathway and IMD pathway differed between *Ae-CLIPB15* and *Ae-CLIPB22* knockdown mosquitoes infected with bacteria and wild type mosquitoes infected with bacteria.

**Conclusions:**

Our findings suggest that *Ae-CLIPB15* and *Ae-CLIPB22* play a critical role in mosquito innate immunity, and that they are involved in immune responses to injury and infection. Their regulation of transcription factors and PO activity indicates that they also play a specific role in the regulation of innate immunity.

**Graphical Abstract:**

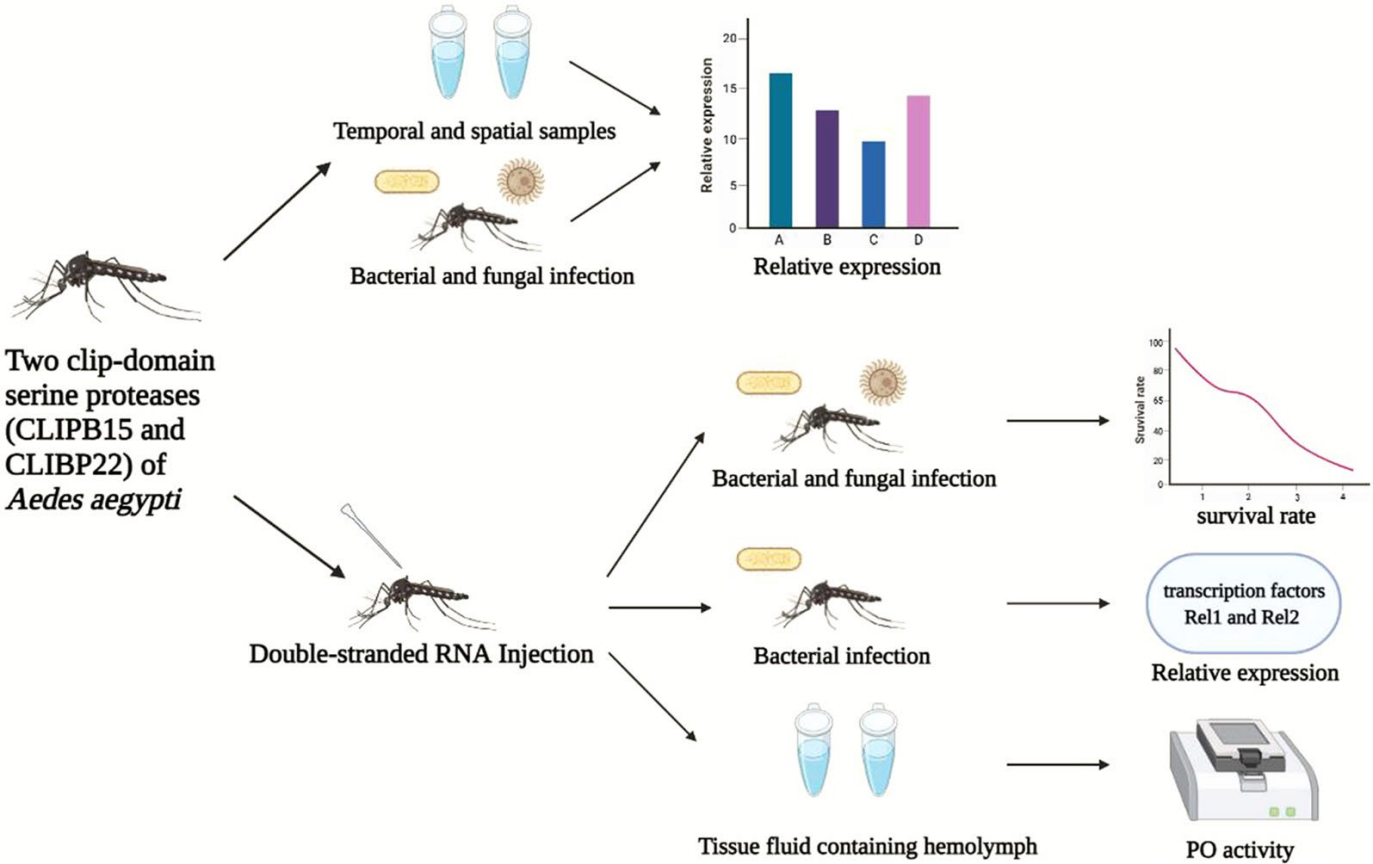

## Background

Mosquito-borne diseases are a major public health problem throughout most of the world [1]. For instance, there are more than 100 million annual cases of dengue fever, a viral disease transmitted by *Aedes aegypti*. Despite the burden of mosquito-borne diseases on human populations, our understanding of the relationships between pathogens and their mosquito hosts remains limited [2]. Studies have shown that innate immunity plays a key role in the interaction between pathogens and their vectors, and that pathogens face multiple barriers in the innate immune system of mosquitoes. Therefore, the study of vector immunity and its interaction with pathogens is important for the development of new vector disease control strategies [3].

Mosquitoes lack acquired immunity, but their innate immune system can destroy various prokaryotic and eukaryotic pathogens [4]. In arthropods, innate immunity plays an important role in limiting pathogen infection through the production of molecules such as antimicrobial peptides (AMPs), through phagocytosis and encapsulation, and by the secretion of physical barriers and melanization [5]. Melanization is an immune response of arthropods to a wide range of viruses [6], bacteria [7, 8], fungi [9], nematodes associated with chromogenic bacteria [10] and other eukaryotic parasites [11–13]. This immune response is regulated by prophenoloxidase (proPO), which mediates the conversion of tyrosine to melanin; recognition of a pathogen triggers a serine protease cascade in which activated serine proteases cleave proPO to produce phenoloxidase (PO) [14]. The Toll pathway and immunodeficiency (IMD) pathway are two major pathways of the innate immune response in arthropods, and are responsible for the production of AMPs and other effectors [15]. In *Ae. aegypti*, the activation of genes coding for AMPs and other immune effectors is achieved by releasing nuclear factor kappa B (NF-κB) transcription factors REL1 and REL2, which are homologous to those in *Drosophila melanogaster* [16].

Clip domain serine proteases (CLIPs), non-digestible serine proteases found in the hemolymph of insects and other arthropods, are key components of the insect immune response. CLIPs have been identified in arthropods and mollusks, and the genes coding for them comprise a large gene family in the insect genome [17–22]. CLIPs are secreted into hemolymph in the form of a zymogen and need to be hydrolyzed and activated by cleavage [14]. CLIPs have a N-terminal with disulfide bonds and a C-terminal domain with protease activity. Infection can stimulate the activation of CLIP protease in hemolymph, which can specifically cleave at a site in the N terminal domain of the protease, resulting in the creation of a double-stranded enzyme in which the CLIP domain and the protease domain are connected by disulfide bonds. Once CLIP proteases are activated, they are regulated by serine inhibitors in hemolymph plasma [23–26]. The functions of CLIPs include proteolytic activation of the Spätzle cytokine to form active Toll ligands for the synthesis of AMPs and the specific activation of proPO that is needed for melanization. However, some CLIPs have a non-catalytic protease-like domain and N-terminal clip domain, which are called clip domain serine protease homologs (CSPHs). Despite their lack of enzymatic activity, CSPHs seem to play an essential role in the immune response of mosquitoes. In melanization, the cleavage and activation of proPO require CSPHs as cofactors [27–29]. Two CSPHs, serine protease homolog (SPH)1 and SPH2 in *Manduca sexta*, are cofactors of proPO activating enzymes (PAP)1 and PAP3, which effectively cleave and activate proPO [29–31]. The precursors of SPH1 and SPH2 cannot activate proPO [29, 32] and need to be treated with PAP3 and PAP1 to generate activated SPH1 and SPH2 [31, 33]. *Tenebrio molitor* CSPH1 [28] and *Holotrichia diomphalia* proPO activating factor II [34] have also been proven to be indispensable CSPHs for the activation of proPO. CSPHs are also involved in cell adhesion and bacterial conditioning [35].

In *Ae. aegypti*, the family of CLIP proteases can be classified into five subfamilies: CLIPA, CLIPB, CLIPC, CLIPD and CLIPE [17]. Of the 82 CLIPs, 62 belong to CLIPB; CLIPC and CLIPD are expected to show serine protease activity. CLIPA and CLIPE comprise 17 CSPHs. In addition, 15 CLIPs and two CSPHs have special domains and are considered to play essential regulatory roles in immune responses.

Through evolutionary analysis we found that there are two members of the CLIPB subfamily in *Ae. aegypti* (Ae): Ae-CLIPB15 and Ae-CLIPB22. These CLIPs are similar to those of other organisms such as *Drosophila melanogaster*, *Bombyx mori* and *Manduca sexta* [3]. However, compared with the CLIPs of different species of mosquitoes, such as *Aedes albopictus* and *Anopheles gambiae*, they are highly conserved, indicating that they may play an important role in the regulation of innate immunity in *Ae. aegypti*. Thus, we decided to examine their role in immune response and immune pathways, and especially their role in the proPO activation pathway. Here, we report the involvement of Ae-CLIPB15 and Ae-CLIPB22 in the defense of *Ae. aegypti* against bacterial and fungal infections. The results showed that these two serine proteases enable *Ae. aegypti* to resist infection by exogenous pathogenic microorganisms. They are also involved in regulating PO activity and transcription factors of innate immunity such as the Toll and IMD pathways. Our study shows that Ae-CLIPB15 and Ae-CLIPB22 are regulatory factors of the mosquito immune system, and thus contributes to a better understanding of the mechanisms of immune regulation in insects.

## Methods

### Mosquito rearing

The *Ae. aegypti* Rockefeller strain was maintained in the insectary at Hainan University. Eggs of *Ae. aegypti* were placed in a 30 × 25 × 8-mm plastic feeding pot and maintained at 27 °C and 90 ± 5% relative humidity. Upon hatching, the larvae were reared in water with fish food provided ad libitum. The adult mosquitoes were kept in the insectary at 26 ± 1 ℃ and 80 ± 5% relative humidity under a 16-h light:18-h dark cycle with access to water and 8% sugar ad libitum. Three-day-old mosquitoes were used for the experiments.

### Multiple amino acid sequence alignment

We retrieved protein sequences of CLIPs from the National Center for Biotechnology Information database (https://www.ncbi.nlm.nih.gov/) that have been studied in other insect species. Clustal Omega software (https://www.ebi.ac.uk/Tools/msa/clustalo/) was used for multiple amino acid sequence alignment of *Ae-CLIPB15* and *Ae-CLIPB22* with different* CLIP*s from other insect species.

### Sampling of different stages and tissue samples of mosquitoes

To examine the temporal expression of *Ae-CLIPB15* and *Ae-CLIPB22*, we used different stages of the mosquito, including eggs, first-instar to fourth-instar larvae, white and black female pupae, white and black male pupae, and female and male adults. To examine the spatial expression of *Ae-CLIPB15* and *Ae-CLIPB22*, different tissues of female adults, including those of the thorax, legs, fat body, midgut, ovary, and Malpighian tubule were collected. Adult female mosquitoes were anesthetized at low temperature and placed in a Petri dish. Each tissue sample comprised material that was collected from about 30 mosquitoes. A total of 50 first- and second-instar larvae and ten larvae at other instar stages were collected from each tube. There were three repeats for each life cycle stage and type of tissue sample.

### Bacterial and fungal infection and tissue injury experiments

For bacterial infection, *Escherichia coli* (DH5α) [36] and *Staphylococcus aureus* (ATCC43300) were obtained from Hainan University. Both were cultured in 37 ℃ Luria–Bertani liquid medium, and their growth monitored by the absorbance reading at 600 nm until the optical density reached about 1. Pellets were collected after centrifugation, then suspended in sterile double-distilled H_2_O to obtain a final *E. coli* cell suspension of 2.0 mg/mL and *S. aureus* cell suspension of 0.8 mg/mL. Adult female mosquitoes were anesthetized on ice for 5 min and then gradually injected with 2 µL of bacterial suspension. The injected adult female mosquitoes were divided into three groups of 30 mosquitoes each for use in the follow-up experiment.

*Beauveria bassiana* (strain 242) was obtained from the microbial bank of Hainan University. *Beauveria bassiana* was cultured on potato glucose agar plates at 28 ℃ for 10 days until the plate was covered with hyphae and conidia. Then eight to 10 fungal colonies were added to 100 ml potato dextrose broth and cultured at 28 ℃, 240 r.p.m. for several days. After the medium was turbid, it was filtered through aseptic neutral filter paper and the filtrate aspirated into aseptic 1.5-ml Eppendorf tubes for centrifugation. The harvested spores were suspended in 0.05% Tween 80. Fungal spores were counted under a hemocytometer with an inverted microscope and diluted to 2.65 × 10^6^ spores/mL with 0.05% Tween 80. An appropriate amount of spore suspension was dipped into a ground aseptic capillary tube, which was used to pierce the ventral tip of the frozen anesthetized adult female mosquitoes, to infect them with *B. bassiana* spores. The infected adult female mosquitoes were divided into three groups comprising 30 mosquitoes each, which were used in the follow-up experiment.

For the tissue injury experiment, the thorax of freeze-anesthetized adult female mosquitoes was punctured with a ground aseptic capillary tube to cause effective physical damage. The adult female mosquitoes were divided into three groups comprising 10 mosquitoes each. Three independent repeated experiments were carried out for each of the above treatments.

### RNA extraction and quantitative polymerase chain reaction

To analyze the spatiotemporal expression profiles of *Ae-CLIPB15* (GenBank accession number AAEL014349) and *Ae-CLIPB22* (GenBank accession number AAEL008668) and their expression profiles after microbial infection and tissue injury, the total RNA was extracted from the different tissues of female adult mosquitoes (those of the thorax, fat body and other parts), and from different developmental stages, as well as from adult female mosquitoes after microbial infection and tissue injury. Total RNA was extracted from adult mosquitoes for each of the three independent repeats. Quantitative real-time polymerase chain reaction (qPCR) was performed as described previously [37] in a LightCycler 480 system (Roche Applied Science, Mannheim, Germany) using SYBR Green Master I (Roche) according to the manufacturer’s instructions, with the following cycling conditions: initial denaturation at 95 ℃ for 30 s followed by 40 cycles of 95 ℃ for 5 s, and 60 ℃ for 30 s. Primers (forward, 5'-CTGTAAGGTCCTGTGAATACG-3'; reverse, 5'-GGTTTATCAGGGAGTTCACC-3') were used to amplify the 109-bp DNA fragment of *Ae-CLIPB15*. Primers (forward, 5'-GATCCTGTCAAAGGCTTCC-3'; reverse, 5'-GTACTGTCAGTGCGTATTGG-3') were used to amplify the 236-bp DNA fragment of *Ae-aaCLIP22*. The* Ae. aegypti* ribosomal protein S17 gene (GenBank accession number AAEL025999) was used as a reference gene. Messenger RNA (mRNA) expression was quantified using the comparative cross threshold (Ct; number of PCR cycles required for the fluorescent signal to cross the signal threshold) method. The relative 2^−ΔΔct^ method was used to analyze the relative gene expression data [38].

### Double-stranded RNA preparation

Total RNA was extracted from adult mosquitoes with the SPARKeasy Cell RNA Kit (Sparkjade) and then subjected to qPCR using the PrimeScript RT Reagent Kit with the genomic DNA Eraser Kit (TaKaRa). Primers (forward, 5'-GGAACTCCCTGATAAACC-3'; reverse, 5'-AACGCACATATTCTAACG-3') were used to amplify the fragment of *Ae-CLIPB15* from the cDNA. Primers (forward, 5'-TGTGGCACTGCTTCCGATTT-3'; reverse, 5'-GAGTATTGCGTAGTCCTTGTA-3') were used to amplify the fragment of *Ae-CLIPB22* from the cDNA. The β-glucuronidase gene (*gus*) (KY848224), a bacterial gene specific to *E. coli*, was used as the negative control, as reported previously [39]. The PCR products were collected and purified. The gene fragments were cloned into PMD19-T (TaKaRa), and NotI and XhoI restriction enzymes used to excise target fragments from PMD19-T, which were then ligated into plasmid pL4440. The recombinant plasmid was transformed into competent cells of the RNase-III-deficient *E. coli* strain HT115(DE3), following a previously described method [39]. The cells were grown in 2× Luria–Bertani medium (10 g/l tryptone, 5 g/l yeast extract, 10 g/l NaCl) containing ampicillin and tetracycline at 37 ℃ for 12–14 h. The bacterial solution was grown until optical density measured at a wavelength of 600 nm reached 0.5. Isopropyl-β-D-thiogalactopyranoside was added to a final concentration of 0.6 mM to induce T7 polymerase activity. The expressed double-stranded RNA (dsRNA) was extracted and confirmed by electrophoresis on 1% agarose gel.

### RNA interference

The adult mosquitoes were collected 3–5 days after hatching and 24 h after they had fed on sugar water. A moderate number of mosquitoes were placed in a refrigerator at − 20 ℃ for a few minutes. The frozen mosquitoes were then put on ice and injected with purified dsRNA by using a manual microinjection device (Eppendorf, Hamburg, Germany). Meanwhile, four groups of females were injected in the thorax with 2 μl of one of the following: the dsRNA of *gus* (control group); diethyl pyrocarbonate (DEPC)-treated water (control group); dsRNA of *Ae-CLIPB15* (ds*Ae-CLIPB15*); dsRNA of *Ae-CLIPB22* (ds*Ae-CLIPB22*). Thirty female adults were injected in each group, and all the experiments were repeated three times. After injection, the mosquitoes were transferred to mosquito cages and collected 24 h later for the preparation of RNA. RNA interference (RNAi) efficiency was verified by qPCR.

### Mosquito survival assays and analysis of transcription factor expression

Twenty-four hours after dsRNA injection, the four groups of mosquitoes were infected with either *E. coli*, *S. aureus*, or *B. bassiana*. Twenty adults from each group were used in the survival assays, which were carried out at 1-day intervals during the following week. Three independent biological repeat experiments were carried out for each treatment. To explore whether the expression of transcription factors *Ae-REL1* (GenBank accession number AAEL012164) and *Ae-REL2* (GenBank accession number AAEL007624) would be affected when *Ae-CLIPB15* and *Ae-CLIPB22* were knocked down in the adults 24 h after dsRNA injection, four groups of mosquitoes were infected with one of the above bacteria. After 24 h, total RNA was extracted for three independent recombination experiments. Primers (forward, 5'-ATAGGCGAGATCAACATCAGCAGC-3'; reverse, 5'-CGTTGCTGTTCCTGCTTCATATCG-3') were used to amplify the fragment of *Ae-REL1*. Primers (forward, 5'-TTTGAATGTGCTGTTGGGTC-3'; reverse, 5'-GAATGTTGTTTCCGTGCTTA-3') were used to amplify the fragment of *Ae-REL2* using cDNA as a template. All the analyses were repeated three times.

### PO activity assay

To determine PO activity, adult mosquitoes were injected with dsRNA 2 days prior to the experiment. The thoraces of ten mosquitoes from each group were added to a 1.5-ml Eppendorf tube with 200 µl HEPES buffer (50 mmol/L), and then crushed with a grinding machine and centrifuged at 12,000 r.p.m. for 20 min at 4 ℃ to collect the hemolymph. A total volume of 75 µl tissue fluid containing hemolymph and 125 µl of L-dopamine (8 mmol/L in 50 mmol/L HEPES, pH 8.0) were added to a 96-well plate and the absorption at 490 nm was recorded every 10 s for 20 min on a microplate reader (RNE90002; Reagen, China). One unit of PO activity is defined as the amount of enzyme yielding PO that produces an increase of 0.001 absorbance units/min [40]. Each treatment was performed as three independent biological replications.

### Statistical analyses

All the statistical analyses were performed using GraphPad Prism version 6.02 (GraphPad Software). A* t*-test was used to determine significant differences (*P* < 0.05) in the levels of mRNA and PO between the control and treatment groups. GraphPad Prism software was used to analyze the mosquito survival curves.

## Results

### Multiple amino acid sequence alignment

**Fig. 1:**
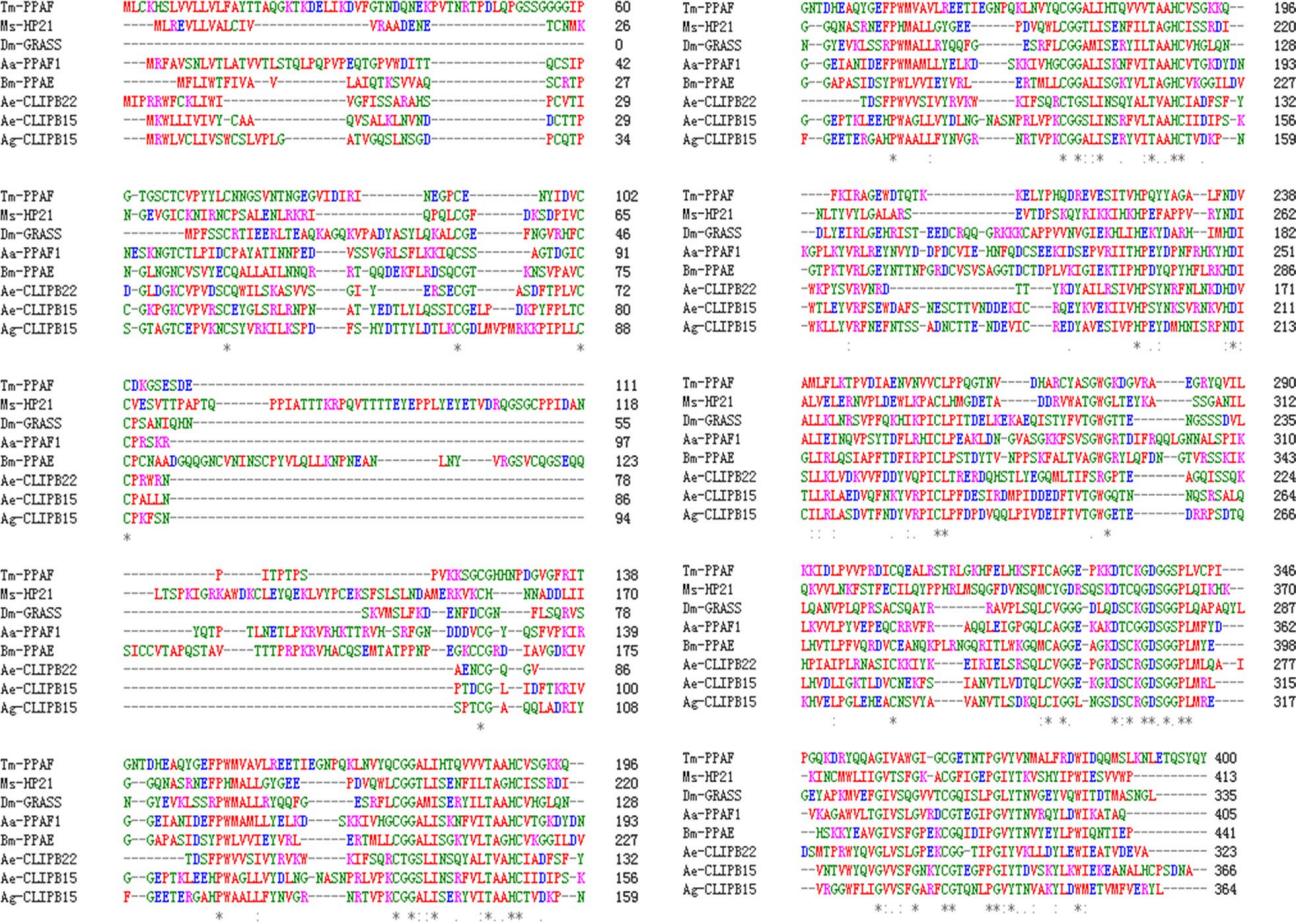
Multiple amino acid sequence alignment of *Aedes aegypti* (Ae) clip domain serine proteases (CLIPs) (*Ae-CLIPB15* and* Ae-CLIPB22*) with CLIPs from other insect species: *Anopheles gambiae* CLIPB15 (*Ag-CLIPB15*, AGAP009844-PA); *Drosophila melanogaster* GRASS (*Dm-GRASS*, Dmel_CG5896); *Manduca sexta* HP21 (*Ms-HP21*, AAV91019.1); *Bombyx mori* prophenoloxidase-activating enzyme (*Bm-PPAE*, NP_001036832.1); *Aedes albopictus* prophenoloxidase activating factor (*Aa-PPAF1*, XP_029722923.1). These sequences were aligned by the Clustal Omega program with default settings. *Asterisks* indicate 100% homology,* colons* indicate 90% homology,* points* indicate 80% homology

Multiple amino acid sequence alignment showed that* Ae. aegypti CLIPB15* is similar to *An. gambiae CLIPB15* (percent identity 49.16%), *Bombyx mori PPAE* (percent identity 37.10%) and *Drosophila melanogaster GRASS* (percent identity 34.20%); and that* Ae-CLIPB22* is similar to *Bombyx mori PPAE* (percent identity 34.53%) and *An. gambiae CLIPB15* (percent identity 34.33%) (Fig. 1).

### Spatiotemporal expression profiles of *Ae-CLIPB15* and *Ae-CLIPB22*

**Fig. 2 a–d:**
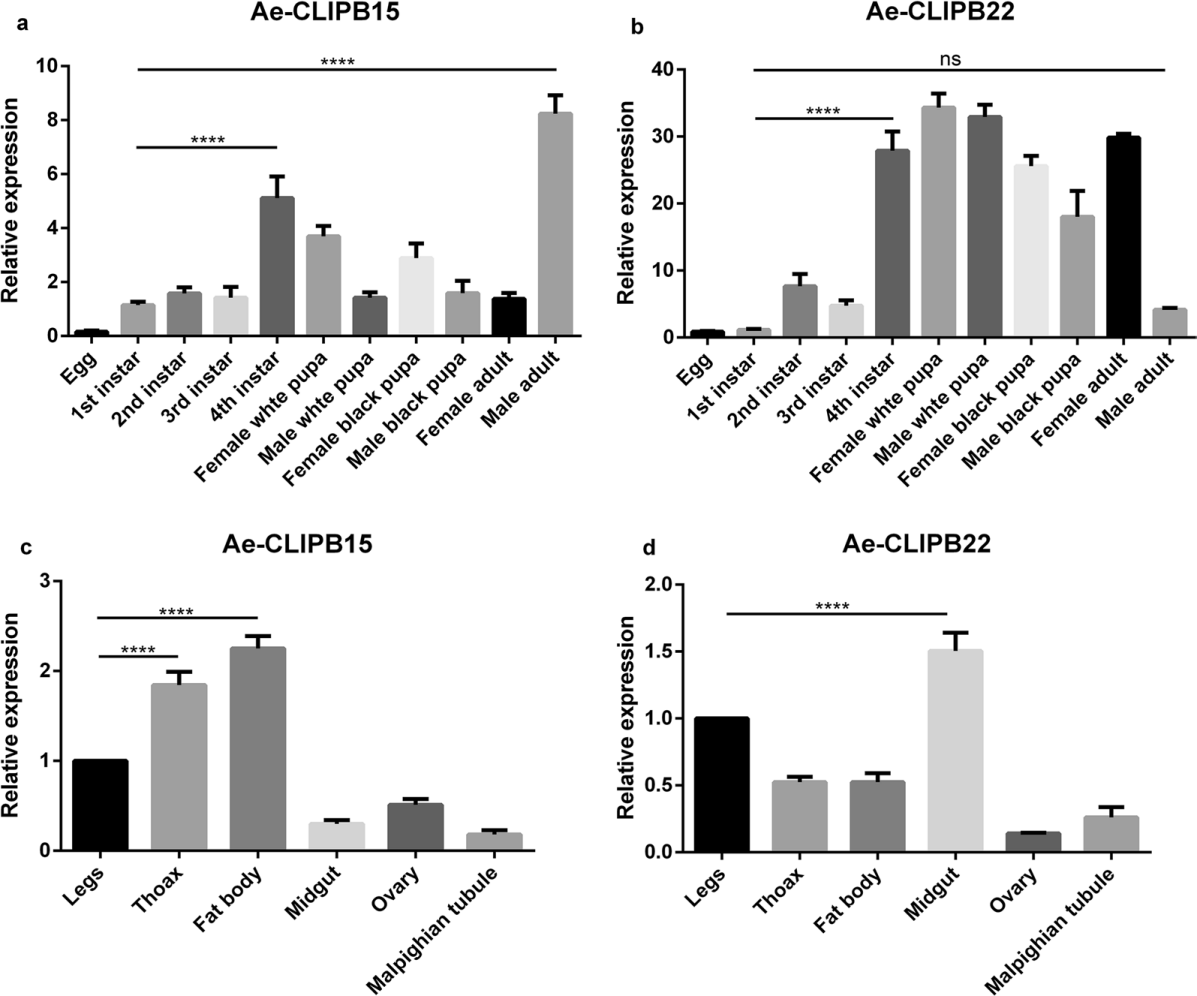
Spatiotemporal expression profiles of *Ae-CLIPB15* and *Ae-CLIPB22*. Relative expression levels of *Ae-CLIPB15* (**a**) and *Ae-CLIPB22* (**b**) at 11 developmental stages from egg to adult. Relative expression levels of *Ae-CLIPB15* (**c**) and *Ae-CLIPB22* (**d**) in six different types of tissues (thorax, legs, fat body, midgut, ovary, and Malpighian tubule). The statistical analyses were performed using two-way ANOVA in GraphPad software; data are presented as means ± SD (*n* = 3). **** *P* < 0.001;* ns* not significantly different. For abbreviations, see Fig.  1

Spatiotemporal expression profiles of *Ae-CLIPB15* and *Ae-CLIPB22* were analyzed by qPCR. The transcript expression levels of these genes were investigated in 11 different developmental stages including the egg, larval, pupal and adult stages, and in tissues of six different parts of the adult mosquito (legs, thorax, fat body, midgut, ovary, and Malpighian tubule). The *Ae-CLIPB15* transcript expression levels in the fourth-instar larvae were significantly higher than in the other larval stages [e.g. first-instar vs fourth-instar, *t*-test, *t*_(22)_ = 11.20, *P* < 0.0001], and those in adult male mosquitoes were significantly higher than in first-instar larvae [first-instar vs male adult, *t*-test, *t*_(22)_ = 20.00, *P* < 0.0001] (Fig. 2a) and in adult females. The transcript expression levels of *Ae-CLIPB22* had significantly increased by the fourth-instar larval stage compared to the first-instar [first-instar vs fourth-instar, *t*-test, *t*_(22)_ = 17.67, *P* < 0.0001] and the higher level was maintained in the pupal and adult female mosquito stages. However, the transcript expression level of this gene decreased significantly in adult male mosquitoes [first-instar vs male adult, *t*-test, *t*_(22)_ = 1.986, *P* = 0.3120] (Fig. 2b). The expression of both genes was lowest in the eggs.

*Ae-CLIPB15* and *Ae-CLIPB22* were expressed in different tissues of adult female mosquitoes. The transcript expression levels of *Ae-CLIPB15* in the thorax [legs vs thorax, *t*-test, *t*_(12)_ = 11.34, *P* < 0.0001] and fat body [legs vs fat body, *t*-test, *t*_(12)_ = 16.80, *P* < 0.0001] were significantly higher than in the other tissues (Fig. 2c). Transcripts of *Ae-CLIPB22* were most abundant in the midgut [legs vs midgut, *t*-test, *t*_(12)_ = 8.583, *P* < 0.0001], compared to the other types of tissues (Fig. 2d).

### Microbial infection and tissue injury can induce the expression of *Ae-CLIPB15* and *Ae-CLIPB22*

**Fig. 3:**
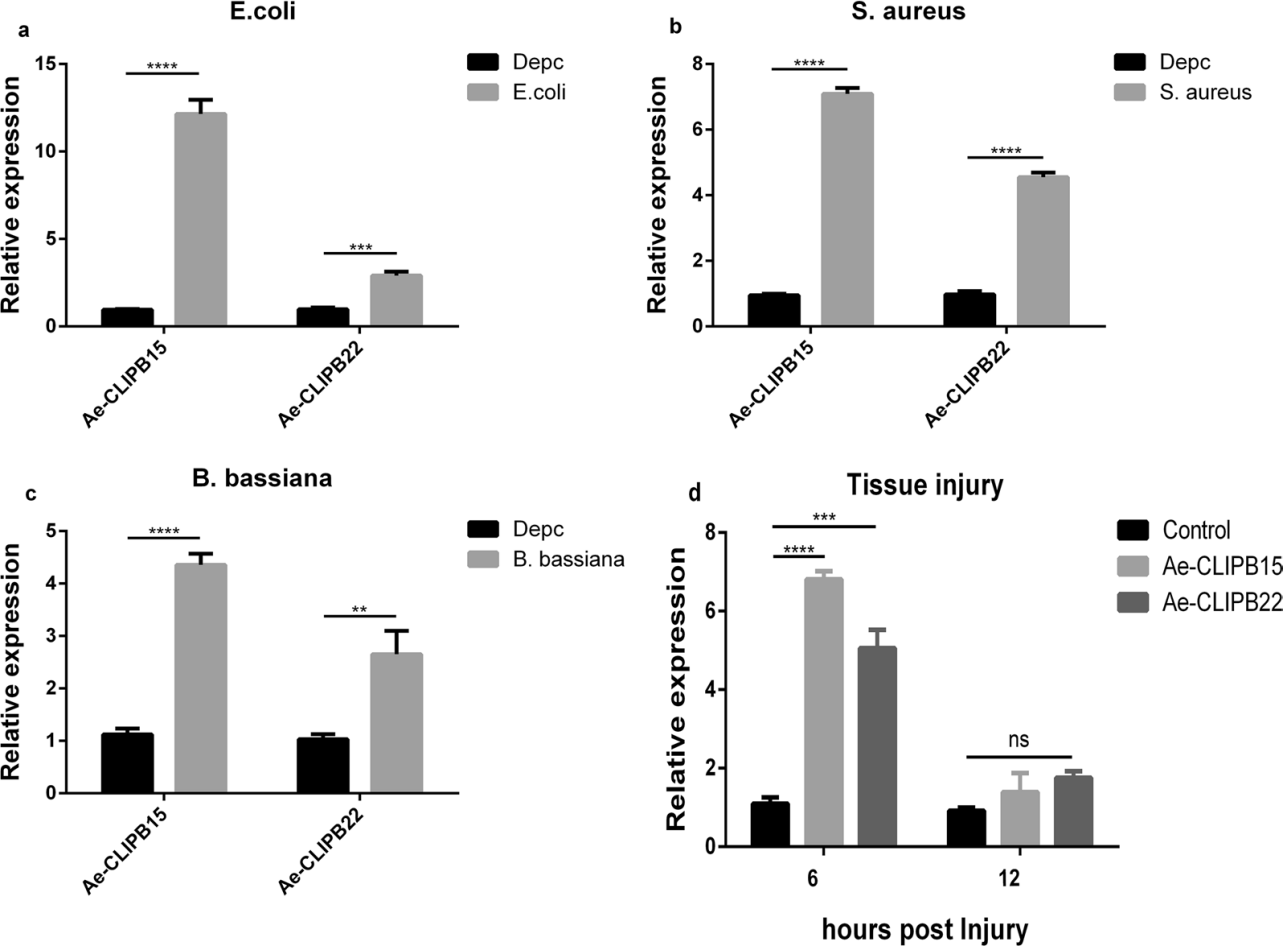
Relative expression levels of *Ae-CLIPB15* and *Ae-CLIPB22* in adult mosquitoes following infection with *Escherichia coli* (**a**), *Staphylococcus aureus* (**b**) and *Beauveria bassiana* (**c**), and after physical injury (**d**). The control groups were injected with diethyl pyrocarbonate (*Depc*)-treated water. The abundances of messenger RNA (mRNA) of *Ae-CLIPB15* and *Ae-CLIPB22* were determined by quantitative real-time polymerase chain reaction (qPCR). The expression of these genes in the infection group was compared to that in the control group at 24 h post-infection. In the physical damage experiment, the control group was not injured. The mRNA abundance for *Ae-CLIPB15* and *Ae-CLIPB22* was determined by qPCR, and the mRNA expression levels of *Ae-CLIPB15* and *Ae-CLIPB22* in the injured groups were compared with those in the control group at 6 h and 12 h post-injury. The expression profiles were compared using two-way ANOVA in GraphPad software; data are presented as means ± SD, *n* = 3. ** *P* < 0.01, *** *P* < 0.001, **** *P* < 0.001. For other abbreviations, see Fig.  1

To determine whether *Ae-CLIPB15* and *Ae-CLIPB22* respond to microbial challenge in *Ae. aegypti*, we first measured their expression levels after bacterial and fungal infection. Infection with the Gram-positive bacterium *S. aureus* and with the Gram-negative bacterium *E. coli* induced up-regulation of *Ae-CLIPB15* [DEPC vs *E. coli*, *t*-test, *t*_(4)_ = 28.66, *P* < 0.0001; DEPC vs *S. aureus*, *t*-test, *t*_(4)_ = 15.71, *P* < 0.0001] and *Ae-CLIPB22* (DEPC vs *E. coli*, *t*-test, *t*_(4)_ = 14.36, *P* = 0.0002; DEPC vs *S. aureus*, *t*-test, *t*_(4)_ = 26.55, *P* < 0.0001] (Fig. 3a, b). Infection with the fungus *B. bassiana* could induce up-regulation of *Ae-CLIPB15* [DEPC vs *B. bassiana*, *t*-test, *t*_(4)_ = 26.99, *P* < 0.0001] and *Ae-CLIPB22* [DEPC vs *B. bassiana*, *t*-test, *t*_(4)_ = 6.109, *P* = 0.0036] (Fig. 3c). Similarly, the expression of *Ae-CLIPB15* [control vs 6 h, *t*-test, *t*_(6)_ = 37.29, *P* < 0.0001] and *Ae-CLIPB22* [control vs 6 h, *t*-test, *t*_(6)_ = 13.87, *P* = 0.0002] was up-regulated 6 h after *Ae. aegypti* mosquitoes were exposed to superficial physical damage (Fig. 3d). *Ae-CLIPB15* and *Ae-CLIPB22* showed different responses to bacterial and fungal infection, and played a specific role in regulating wound healing in the mosquitoes.

### Knockdown of *Ae-CLIPB15* and *Ae-CLIPB22* resulted in higher susceptibility to bacterial and fungal infections

**Fig. 4:**
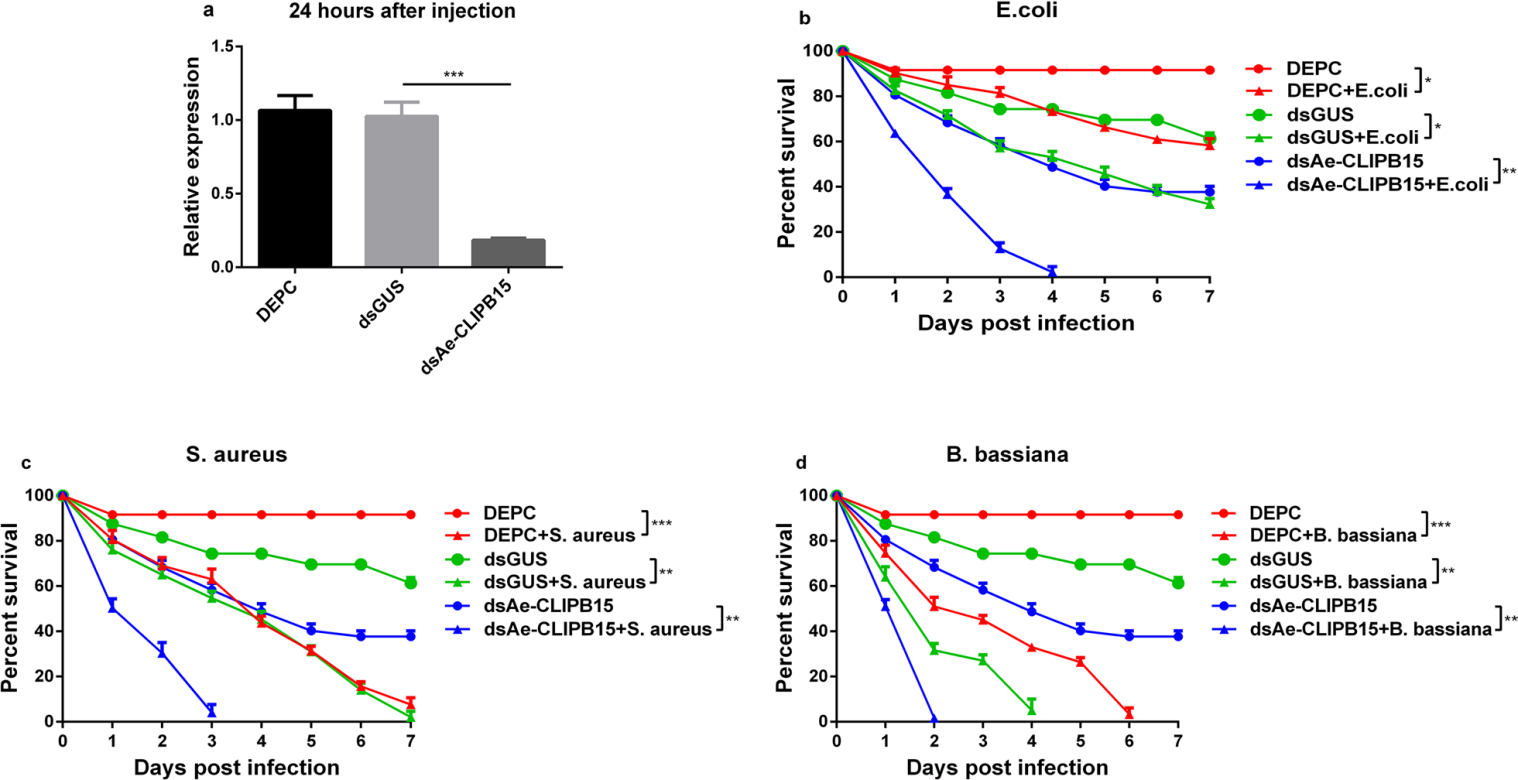
Knockdown of *Ae-CLIPB15* by RNA interference (RNAi) (**a**) and its effect on the survival of adult mosquitoes after *Escherichia coli* (**b**), *Staphylococcus aureus* (**c**) and *Beauveria bassiana* (d) infection. The mRNA expression levels of *Ae-CLIPB15* were examined by qPCR after double-stranded (ds) RNA injection (*dsAe-CLIPB15*). The expression profiles were compared using two-way ANOVA in GraphPad software; data are presented as means ± SD, *n* = 3. * *P* < 0.05, ** *P* < 0.01, *** *P* < 0.001.* GUS* β-Glucuronidase; for other abbreviations, see Figs.  1 and 3

**Fig. 5:**
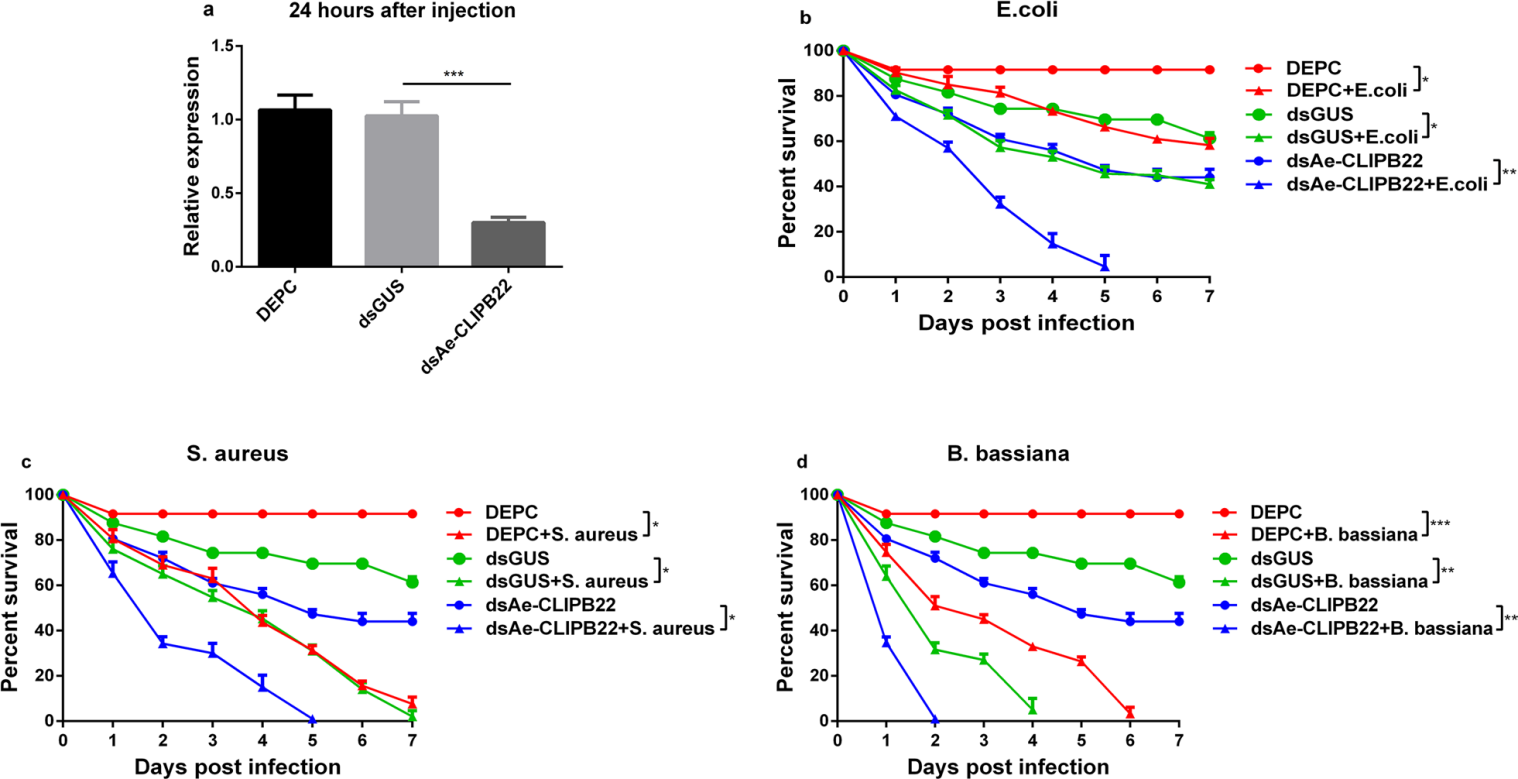
Knockdown of *Ae-CLIPB22* by RNAi (**a**) and its effect on the survival of adult mosquitoes after *Escherichia coli* (**b**), *Staphylococcus aureus* (**c**) and *Beauveria bassiana* (**d**) infection. The mRNA expression levels of *Ae-CLIPB22* were examined by qPCR after dsRNA injection (*dsAe-CLIPB22*). The expression profiles were compared using two-way ANOVA in GraphPad software; data are presented as means ± SD, *n* = 3. * *P* < 0.05, ** *P* < 0.01, *** *P* < 0.001. For abbreviations, see Figs.  1, 3 and  4

To confirm whether *Ae-CLIPB15* and *Ae-CLIPB22* contribute to the defense of *Ae. aegypti* mosquitoes against bacterial and fungal infection, we injected dsRNA into the mosquito thorax and monitored the survival of the female adults after infection. At 24 h post-infection, the relative mRNA expression levels of *Ae-CLIPB15* [GUS vs dsAe-CLIPB15, *t*-test, *t*_(6)_ = 12.76, *P* < 0.0001] and *Ae-CLIPB22* [GUS vs dsAe-CLIPB22, *t*-test, *t*_(6)_ = 14.80, *P* < 0.0001] were significantly decreased, indicating successful knockdown of these two genes (Figs. 4a, 5a). The female adults were also infected with bacteria and fungi after successful *Ae-CLIPB15* and *Ae-CLIPB22* knockdown. Knockdown of *Ae-CLIPB15* [dsGUS + *E. coli* vs dsAe-CLIPB15 + *E. coli*, *t*-test, *t*_(12)_ = 3.251, *P* = 0.0314; dsGUS + *S. aureus* vs dsAe-CLIPB15 + *S. aureus*, *t*-test, *t*_(12)_ = 5.547, *P* = 0.0149] and *Ae-CLIPB22* [dsGUS + *E. coli* vs dsAe-CLIPB22 + *E. coli*, *t*-test, *t*_(12)_ = 3.333, *P* = 0.0207; dsGUS + *S. aureus* vs dsAe-CLIPB22 + *S. aureus*, *t*-test, *t*_(12)_ = 4.803, *P* = 0.0187] resulted in a significant decrease in the survival rate of mosquitoes infected with *E. coli* (Figs. 4b, 5b) and *S. aureus* (Figs. 4c, 5c) compared with the control groups. The survival rate of mosquitoes infected with *B. bassiana* [dsGUS + *B. bassiana* vs dsAe-CLIPB15 + *B. bassiana*,* t*-test, *t*_(12)_ = 8.71, *P* = 0.0074; dsGUS + *B. bassiana* vs dsAe-CLIPB22 + *B. bassiana*,* t*-test, *t*_(12)_ = 7.251, *P* = 0.0102] was also significantly lower than that of the control group (Figs. 4d, 5d). In summary, these results show that both of these genes are implicated in immune reactions triggered in response to bacterial and fungal infection.

### Knockdown of *Ae-CLIPB15* and *Ae-CLIPB22* resulted in different regulation of transcription factors

**Fig. 6:**
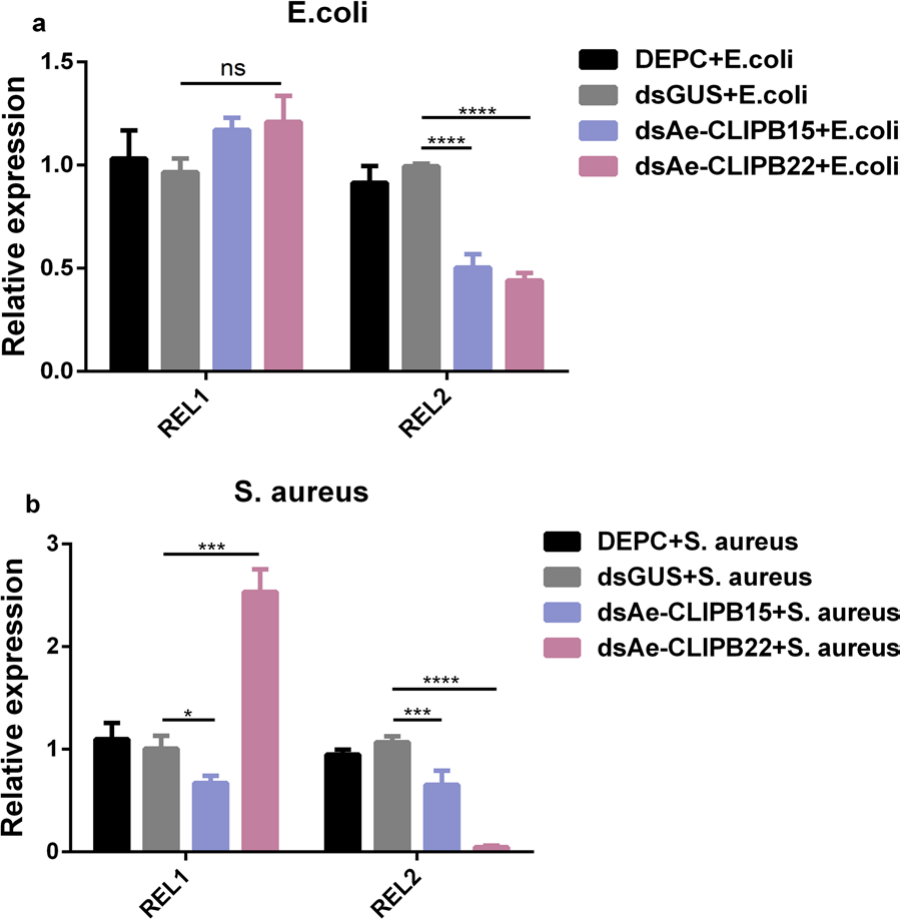
Knockdown of *Ae-CLIPB15* and *Ae-CLIPB22* by RNAi and the effect on the expression levels of NF-κB transcription factors *REL1* (**a**) and *REL2* (**b**) in the Toll pathway and IMD pathway of mosquitoes after bacterial infection. The relative expression levels of *REL1* and *REL2* were examined by qPCR after bacterial infection. The expression profiles were compared using two-way ANOVA in GraphPad software; data are presented as means ± SD, *n* = 3. * *P* < 0.05, *** *P* < 0.001, **** *P* < 0.001. For other abbreviations, see Figs.  1, 3,  4 and 5

To confirm whether *Ae-CLIPB15* and *Ae-CLIPB22* can regulate the Toll pathway and IMD pathway of the innate immune response of *Ae. aegypti*, the expression levels of NF-κB transcription factors *REL1* and *REL2* in the Toll pathway and IMD pathway were investigated in mosquitoes infected with Gram-negative *E. coli*. *Ae-CLIPB15* and *Ae-CLIPB22* were knocked down by injected dsRNA. The mosquitoes were infected with *E. coli* and *S. aureus* 24 h after dsRNA injection. At 12 h post-infection, total RNA was extracted and qPCR performed. The knockdown of *Ae-CLIPB15* [dsGUS + *E. coli* vs dsAe-CLIPB15 + *E. coli*, *t*-test, *t*_(8)_ = 10.81, *P* < 0.0001] and *Ae-CLIPB22* [dsGUS + *E. coli* vs dsAe-CLIPB22 + *E. coli*, *t*-test, *t*_(8)_ = 12.19, *P* < 0.0001] resulted in a significant decrease in the transcript expression levels of NF-κB transcription factor *REL2* in the IMD pathway after the female adults were infected by the Gram-negative bacteria *E. coli*. In contrast, the expression of NF-κB transcription factor *REL1* in the Toll pathway was not significantly different from that in the control group (Fig. 6a). Interestingly, when the female adults were infected with the Gram-positive bacteria *S. aureus*, the expression of NF-κB transcription factor *REL1* in the Toll pathway was significantly decreased in the *Ae-CLIPB15* knockdown adults [dsGUS + *S. aureus* vs dsAe-CLIPB15 + *S. aureus*, *t*-test, *t*_(8)_ = 3.233, *P* = 0.0298] but significantly increased in *Ae-CLIPB22* knockdown adults [dsGUS + *S. aureus* vs dsAe-CLIPB22 + *S. aureus*, *t*-test, *t*_(8)_ = 12.36, *P* < 0.0005]. The expression of NF-κB transcription factor *REL2* in the IMD pathway was significantly decreased in *Ae-CLIPB15* knockdown adults [dsGUS + *S. aureus* vs dsAe-CLIPB15 + *S. aureus*, *t*-test, *t*_(8)_ = 6.411, *P* = 0.0005] and *Ae-CLIPB22* knockdown adults [dsGUS + *S. aureus* vs dsAe-CLIPB22 + *S. aureus*, *t*-test, *t*_(8)_ = 15.83, *P* < 0.0001] (Fig. 6b). Although the expression of *REL1* was not affected by the knockdown of either *Ae-CLIPB15* or *Ae-CLIPB22* in mosquitoes infected with *E. coli*, the knockdown of these genes did affect its expression in the mosquitoes infected with *S. aureus*. In short, these results show that both *Ae-CLIPB15* and *Ae-CLIPB22* are involved in the regulation of the expression of *REL1* and *REL2* in the Toll pathway and the IMD pathway in the innate immune response of *Ae. aegypti*, and suggest that these genes play different roles in the regulation of innate immune pathways. However, the differences in the expression level of REL2 do not necessarily mean that *Ae-CLIPB15* and *Ae-CLIPB22* directly regulate the IMD pathway, as innate immune pathways may interact with each other. Thus, the change in the expression of REL2 may have been caused by an indirect effect. In summary, further experiments are needed to examine this.

### Knockdown of *Ae-CLIPB15* decreased proPO activation in *Ae. aegypti*

**Fig. 7:**
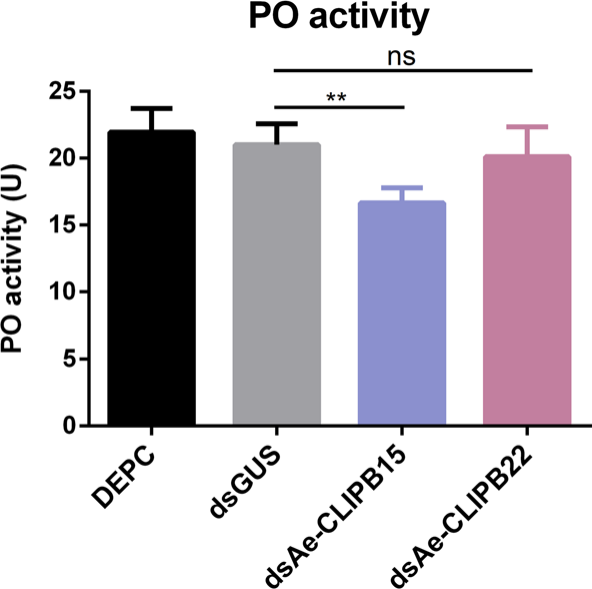
Phenoloxidase (*PO*) activity of knockdown *Ae-CLIPB15* and *Ae-CLIPB22* mosquitoes. Adult mosquitoes were injected with dsRNA and the hemolymph of 10 mosquitoes was collected 24 h later for PO activity assays on a microplate reader. PO activities were compared using two-way ANOVA in GraphPad software; data are presented as means ± SD, *n* = 3. ** P < 0.01. For other abbreviations, see Figs.  1, 3,  4 and 5

The change in PO activity in the *Ae-CLIPB15* and *Ae-CLIPB22* knockdown adults was examined in the hemolymph at 24 h post-injection. Compared with the control group, the knockdown of *Ae-CLIPB15* [dsGUS vs dsAe-CLIPB15, *t*-test, *t*_(12)_ = 3.765, *P* = 0.0056] resulted in a significant decrease in PO activity. However, there was no significant difference in PO activity following the knockdown of *Ae-CLIPB22* (dsGUS vs dsAe-CLIPB22, *t*-test, *t*_(12)_ = 0.7704, *P* = 0.7839] (Fig. 7). In general, the PO activity data showed that *Ae-CLIPB15* is involved in the activation of proPO in *Ae. aegypti*, while *Ae-CLIPB22* is not.

## Discussion

Insects rely on their innate immune system to fight invading bacteria, fungi and parasites [16, 41–44]. The innate immune system plays an important role in limiting pathogen infection through phagocytosis, entrapment, secretion of physical barriers and melanization. These innate immune responses are mainly studied using insect models. CLIPs are essential components of the insect immune response. The CLIP catalytic domain is characterized by a conserved Tryp_SPc domain with a catalytic ternary domain, which is composed of the amino acid residues His, Asp and Ser [45, 46]. When the catalytic triad of SP mutates, the enzyme loses its catalytic activity and becomes a CSPH. The functions of CLIPs in innate immune responses include the proteolytic activation of the cytokine Spätzle to form active Toll ligands for the synthesis of AMPs and the specific activation of proPO required for melanization. In *Ae. aegypti*, the CLIP family is classified into five subfamilies—CLIPA, CLIPB, CLIPC, CLIPD, CLIPE—each of which comprises polygenes. In this study, we were able to confirm the roles of *Ae-CLIPB15* and *Ae-CLIPB22*, two members of the CLIPB subfamily of *Ae. aegypti*, in the innate immune response of adult *Ae. aegypti* mosquitoes.

The qPCR results showed that the kinetics of the induction of *Ae-CLIPB15* and *Ae-CLIPB22* differ following infection by Gram-negative and Gram-positive bacteria and fungi, but that these genes are not co-regulated. Interestingly, *Ae-CLIPB15* and *Ae-CLIPB22* are also induced by aseptic injury. Our results suggest that *Ae-CLIPB15* and *Ae-CLIPB22* may play a role in microbial defense and potentially a regulatory role in wound healing.

The immune response of arthropods is mainly regulated by two pathways, the Toll pathway and the IMD pathway [15]. In *Ae. aegypti*, NF-κB transcription factors *REL1* and *REL2* of the Toll pathway and IMD pathway are the main participants in the activation of genes coding for AMPs and other immune effector factors. Spz1C, Toll5A, CLIPB5 and CLIPB29 of *Ae*. *aegypti* have been shown to mediate the response of the Toll pathway to fungal infection [3, 47]. In *An. gambiae*, the IMD pathway is activated by peptidoglycan recognition protein or indirectly activated by a serine protease cascade [48]. However, according to current knowledge of insect immunity, CLIPs are not involved in the IMD pathway. Here, we used RNAi to knock down the dsRNA expression of *Ae-CLIPB15* and *Ae-CLIPB22* to study their role in the innate immunity of *Ae. aegypti*. After *Ae-CLIPB15* and *Ae-CLIPB22* were knocked down, the survival rate of *Ae. aegypti* was reduced after infection with pathogenic bacteria compared with that of wild type mosquitoes, and their resistance to bacterial infection decreased by 50%.

The transcription of *REL1* and *REL2* was affected to varying degrees when *Ae-CLIPB15* and *Ae-CLIPB22* were knocked down. However, as the expression of *REL1* and *REL2* was mostly inhibited following *Ae-CLIPB15* and *Ae-CLIPB22* knockdown, we suggest that these latter genes are involved in the regulation of the Toll pathway and have an indirect effect on the activation of the IMD pathway. This may also explain why the survival rate of the adult mosquitoes that had been infected with bacteria and fungi after *Ae-CLIPB15* and *Ae-CLIPB22* knockdown was lower than that of the wild type mosquitoes. However, further biochemical study is definitely needed to examine whether *Ae-CLIPB15* and *Ae-CLIPB22* are involved in the regulation of the Toll pathway or the IMD pathway. In arthropods, the central component of the extracellular enzyme cascade is a CLIP which regulates various innate immune responses such as the Toll pathway and the IMD pathway [42]. Our results confirm that *Ae-CLIPB15* and *Ae-CLIPB22* participate in the regulation of innate immune responses in *Ae. aegypti*; however, their role in the regulation of a serine protease cascade remains to be clarified.

Mosquitoes that are capable of melanization initially received a great deal of attention as specific phenotypes that are resistant to parasites of public health importance, such as those that cause malaria and filariasis [49–51]. Melanization is a powerful defense response in *Ae. aegypti*. In this process, the PO activation system is triggered by soluble receptor molecules that recognize molecular patterns associated with pathogens or abnormal cells, resulting in the activation of serine proteases. These then activate a CLIPC, which in turn activates the terminal CLIPB protease in the cascade, also known as proPO activating protease (PAP) or proPO activating enzyme (PPAE). The activated PAP then cleaves the proPO to give PO. PO then acts as a catalyst for the formation of active intermediates of a quinone for the synthesis of melanin [52], which has a variety of protective functions. In addition, the formation of an active PO complex on the surface of the foreign body is mediated by one or more proteolytic inactivated CLIPAs, which need to be activated through proteolysis to play a role in the process. However, not all the CLIPs involved in the regulation of the proPO cascade activate proPO. Some CLIPs can inhibit melanization in *An. gambiae* [53]. Under normal physiological conditions, the proPO activation cascade is turned off, mainly by a single highly conserved serine protease inhibitor: Spn27A in *Drosophila melanogaster* [54, 55], Serpin-3 in *Manduca sexta* [56], and SRPN2 in mosquitoes [3, 17, 18, 57]. In this study, we knocked down *Ae-CLIPB15* and *Ae-CLIPB22* and explored their role in the proPO activation system. The knockdown of *Ae-CLIPB15* led to a significant decrease in PO activity in the mosquitoes. However, there was no significant difference in PO activity in *Ae-CLIPB22* knockdown mosquitoes. *Ae-CLIPB15* was involved in the regulation of PO activity in *Ae. aegypti*, while *Ae-CLIPB22* was not. This suggests that the CLIP family comprises serine proteases that are involved in a complex network of regulatory cascades that are independent of each other yet show some similarities. Further research is needed on this.

In summary, CLIPs are essential regulators of immune responses in many insects. All presently identified proteases that directly cleave insect proPO belong to the CLIPB subfamily. However, it is not clear how a single melanization cascade is regulated in the context of numerous physiological functions. Deciphering the regulatory mechanisms that lead to melanization in different defense responses, such as wound healing and pathogen isolation, is of particular interest. CLIPs may also have non-immune functions during insect development, e.g. embryonic dorsal pattern formation in *Drosophila*, and play a role in other physiological systems, all of which remain to be discovered [58]. To further improve our understanding of CLIP cascades and their role in a variety of immune responses, we also need to study other organisms, especially arthropod vectors, which show complex interactions between their immune systems and the pathogens that they transmit.

## Conclusions

This study provides evidence for the role of *Ae-CLIPB15* and *Ae-CLIPB22* in the innate immune regulation of mosquitoes. Genetic interference of *Ae-CLIPB15* and *Ae-CLIPB22* could affect the resistance of adult *Ae. aegypti* to pathogens. However, further biochemical study is definitely needed to explore the regulatory mechanisms of innate immunity in *Ae. aegypti*. Our results suggest that *Ae-CLIPB15* and *Ae-CLIPB22* are potential targets for vector transmission control using RNAi technology.

## Data Availability

The data supporting the findings of this article are included in the article and its additional files.

## Abbreviations

AMP: Antimicrobial peptide
cDNA: Complementary DNA
CLIP: Clip domain serine protease
CSPH: Clip domain serine protease homolog
DEPC: Diethyl pyrocarbonate
dsRNA: Double-stranded RNA
GUS: β-Glucuronidase
IMD: Immunodeficiency
mRNA: messenger RNA
PAP: Prophenoloxidase activating enzyme
PO: Phenoloxidase
proPO: Prophenoloxidase
qPCR: Quantitative real-time polymerase chain reaction
RNAi: RNA interference
SPH: Serine protease homolog
